# Real-Time Molecular Monitoring in Acute Myeloid Leukemia With Circulating Tumor DNA

**DOI:** 10.3389/fcell.2020.604391

**Published:** 2020-12-11

**Authors:** Deepshi Thakral, Ritu Gupta, Ranjit Kumar Sahoo, Pramod Verma, Indresh Kumar, Sangeeta Vashishtha

**Affiliations:** ^1^Laboratory Oncology Unit, Dr. BRA IRCH, All India Institute of Medical Sciences, New Delhi, India; ^2^Department of Medical Oncology, Dr. BRA IRCH, All India Institute of Medical Sciences, New Delhi, India

**Keywords:** plasma cell free DNA, cfDNA, circulating tumor DNA, ctDNA, acute myeloid leukemia, genomic profiling, measurable residual disease, hematological malignancies

## Abstract

The clonal evolution of acute myeloid leukemia (AML), an oligoclonal hematological malignancy, is driven by a plethora of cytogenetic abnormalities, gene mutations, abnormal epigenetic patterns, and aberrant gene expressions. These alterations in the leukemic blasts promote clinically diverse manifestations with common characteristics of high relapse and drug resistance. Defining and real-time monitoring of a personalized panel of these predictive genetic biomarkers is rapidly being adapted in clinical setting for diagnostic, prognostic, and therapeutic decision-making in AML. A major challenge remains the frequency of invasive biopsy procedures that can be routinely performed for monitoring of AML disease progression. Moreover, a single-site biopsy is not representative of the tumor heterogeneity as it is spatially and temporally constrained and necessitates the understanding of longitudinal and spatial subclonal dynamics in AML. Hematopoietic cells are a major contributor to plasma cell-free DNA, which also contain leukemia-specific aberrations as the circulating tumor-derived DNA (ctDNA) fraction. Plasma cell-free DNA analysis holds immense potential as a minimally invasive tool for genomic profiling at diagnosis as well as clonal evolution during AML disease progression. With the technological advances and increasing sensitivity for detection of ctDNA, both genetic and epigenetic aberrations can be qualitatively and quantitatively evaluated. However, challenges remain in validating the utility of liquid biopsy tools in clinics, and universal recommendations are still awaited towards reliable diagnostics and prognostics. Here, we provide an overview on the scope of ctDNA analyses for prognosis, assessment of response to treatment and measurable residual disease, prediction of disease relapse, development of acquired resistance and beyond in AML.

## Introduction

Acute myeloid leukemia (AML) is a rapidly progressing hematological malignancy that display varied clinicopathological features as well as treatment outcomes (Estey and Döhner, 2006; Döhner et al., 2015; De Kouchkovsky and Abdul-Hay, 2016). With an annual incidence of 4.3 per 100,000 (age-adjusted cases from 2013 to 2017), the median age at diagnosis of AML is 68 years and 5-year relative survival is 28.7% in the United States alone [National Cancer Institute, 2017; SEER Cancer Stat Facts: Acute Myeloid Leukemia (accessed at https://seer.cancer.gov/statfacts/html/amyl.html; on 20th August, 2020)]. The expanding knowledge of its genomics has revealed the molecular complexity of the abnormal leukemogenesis in AML, which has immensely contributed to the refinement of risk stratification and personalized therapeutic strategies for these patients (Welch et al., 2012; Ley, 2013; Papaemmanuil et al., 2016; Leisch et al., 2019). Of the several novel treatment options approved by the U.S. Food and Drug Administration (FDA) that target specific gene mutations, surface markers, or regulators of apoptosis, epigenetic, or micro-environmental pathways; eight of these drugs have recently been incorporated into clinical practice to guide patient-specific treatment in AML in addition to the standard chemotherapy (Tiong and Wei, 2019; Green and Konig, 2020). Nevertheless, the long-term survival is less than 30% in patients below the age of 60 years and worse in older AML patients with comorbidities (Appelbaum et al., 2006; Almeida and Ramos, 2016). A significant proportion of AML patients eventually encounter disease relapse or become refractory even after initially achieving complete remission (CR) post-induction chemotherapy (Schlenk et al., 2017; Shlush et al., 2017).

In the current scenario with several options of standard chemotherapy, low-intensity regimens, combination chemoimmunotherapy, and clinical trials, AML patients may certainly benefit from the provision of concurrent real-time longitudinal monitoring of disease burden. The persistence of patient-specific genetic alterations in the due course of disease progression holds potential prognostic value. Monitoring these alterations may further guide strategies for maintenance of remission in the long term. Since liquid biopsy techniques are extensively being explored as non-invasive methods for tumor diagnosis and disease monitoring, these can provide early insights into treatment efficacy as well as predict recurrence of the disease. Here, we present an overview of the scope of molecular analysis of ctDNA in tracking relevant genetic markers for longitudinal monitoring of disease burden in AML.

## Transforming Classification of AML Post-Genomics Era

**FIGURE 1:**
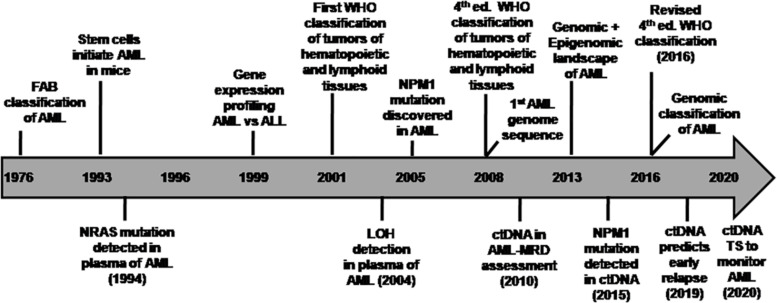
Timeline of various milestones in the evolution of genomic classification of AML (Adapted from TCGA project Ley et al., 2013) and studies on circulating tumor DNA in AML. Initially, AML was classified by the French-American-British (FAB) Cooperative Group (1976) based on the cell lineage of leukemic cells and their differentiation status derived from the cell morphology as well as cytochemical staining of BM cells (Bennett et al., 1976). This approach had limitations in risk stratification in majority of AML cases. The discovery of recurrent cytogenetic abnormalities led to a novel classification system by the World Health Organization [WHO] (2001), which was revised in 2008 and later in the 2016 WHO Classification. Few research highlights of various studies on ctDNA analysis in AML disease monitoring are indicated in the timeline as a co-evolution with advances in AML genomics. ALL, acute lymphoblastic leukemia; AML, acute myeloid leukemia; ctDNA, circulating tumor-derived DNA; ed., edition; LOH, loss of helerozygosity; MRD, measurable residual disease; TS, targeted sequencing; WHO, World Health Organization.

Ever since the World Health Organization (WHO) introduced a new classification system of AML based on recurrent cytogenetic abnormalities in 2001 (Jaffe et al., 2001; Vardiman et al., 2002) and subsequently revised it in 2008 (Vardiman et al., 2009; Swerdlow et al., 2017) and further refined it based on molecular alterations in 2016 (Swerdlow et al., 2016, 2017), AML genome sequencing has further revealed a wide spectrum of driver and co-occurring mutations in majority of the patients (Ley et al., 2008; Mardis et al., 2009). The advent of next-generation sequencing (NGS) has deciphered the heterogeneous molecular complexity of AML genome specifically with cytogenetically normal karyotype (CN-AML) (Welch et al., 2012; Ley et al., 2013; Martelli et al., 2013). This category of CN-AML comprises almost half of the newly diagnosed AML cases (Ghanem et al., 2012; Martelli et al., 2013). In addition to the abnormal myeloid differentiation (gene rearrangements in *RUNX1, CBFB*, *RARA*, and alterations in other transcription factors), it has become evident that more than 95% of AML patients carry known driver and co-occurring mutations that are implicated in the survival of the neoplastic clone or subclones (Gupta, 2016; Papaemmanuil et al., 2016). Somatic mutations became apparent as an independent prognostic factor for risk stratification in CN-AML (Grimwade et al., 2016). Henceforth, WHO classification was updated by incorporating subcategories based on recurrent cytogenetic abnormalities and mutations such as AML with mutated *NPM1* and *CEBPA* as full entities and mutated *RUNX1* as provisional entity in the revised 2016 classification (Arber et al., 2016). The timeline of various milestones highlighting the transition toward genomic classification of AML is shown in Figure 1.

## Functional Genomic Landscape and Risk Assessment in AML

Next-generation sequencing of AML genome has revealed the gene mutation map of driver events that led to a genomic classification of AML with significant clinical correlation (Ley et al., 2013; Papaemmanuil et al., 2016; Bullinger et al., 2017). These mutated genes are now segregated into nine categories based on their biological function (Table 1). Data from the mutational profiling of AML patients by either whole-genome or whole-exome sequencing was obtained by The Cancer Genome Atlas (TCGA) project (Ley et al., 2013). This study documented an average number of 13 coding mutations (single nucleotide variations and insertions/deletions) per patient suggesting a coordinated interaction of these gene alterations in AML pathogenesis (Grimwade et al., 2016). An average of less than one gene fusion event and a median of one somatic copy-number variant (e.g., trisomies or monosomies) were few other findings. Moreover, AML genomes with complex cytogenetic profile strongly associated with mutations in *TP53* gene. The updated European Leukemia Net (ELN) recommendation for risk stratification of AML patients into favorable, intermediate, and adverse groups is governed by a comprehensive integrated genomic profiling that includes recurrent chromosomal abnormalities, gene mutations, and *FLT3-ITD* allelic ratio (Döhner et al., 2017). Currently, most AML cases are assigned prognostically distinct subcategories based on the considerations of clinical presentation and presence or co-occurrence of cytogenetic and molecular aberrations (Hou et al., 2014). Comprehensive molecular profiling is gradually becoming the mainstay for improved diagnosis, prognostication, measurable residual disease (MRD) assessment, eligibility for targeted therapy, and monitoring for better patient management in AML (Pollyea, 2018; Schuurhuis et al., 2018).

**TABLE 1:** Functional categorization of common genetic alterations and their clinical significance in AML (Adapted from Ley et al., 2013; Thakral and Gupta, 2019).

Functional category	Genetic alteration	Functional outcome	Clinical significance/risk stratification
**Class I** Transcription factor fusions by gene rearrangements	*t(8;21)* *inv16/t(16;16)*	Transcriptional deregulation and impaired hematopoietic differentiation	Favorable risk (absence of KIT*/FLT3*)
**Class II** Myeloid transcription factors	*CEBPA* *RUNX1* *GATA2*	Transcriptional deregulation and impaired hematopoietic differentiation	Intermediate to adverse risk
**Class III** Nucleophosmin 1	*NPM1*	Aberrant cytoplasmic localization of NPM1	Favorable risk (absence of FLT3*)
**Class IV** Tumor suppressor	*TP53* *PHF6* *WT1*	Transcriptional deregulation and impaired degradation	Adverse prognosis
**Class V** Signaling genes	*FLT3* *KIT*	Proliferative advantage conferred by activation of RAS/RAF, JAK/STAT, and PI3K/AKT signaling pathways	Midostaurin, a multikinase inhibitor approved by FDA for oral use.
	*KRAS/NRAS*		Adverse prognosis in CBF AML
	*PTPN11*		Diagnostic markers
**Class VI** DNA methylation-associated genes	*DNMT3A* *DNMT3B*	Deregulation of DNA methylation, transcriptional deregulation	Adverse prognosis in CN-AML
	*IDH1* *IDH2*		IDH1/2 anti-metabolite enzyme inhibitors
	*TET1* *TET2*		Associated with adverse prognosis
**Class VII** Chromatin modifiers	*ASXL1* *EZH2* *KMT2A* fusions *KMT2A*-PTD	Deregulation of chromatin modification and transcriptional deregulation	Adverse prognosis
**Class VIII** Spliceosome-complex genes	*SRSF2* *SF3B1* *U2AF1* *ZRSR2*	Impaired spliceosome function; aberrant splicing	Adverse prognosis
**Class IX** Cohesin complex genes	*RAD21* *SMC1/SMC2* *STAG2*	Possible impairment of chromosome segregation and transcriptional regulation	Under investigation

*Mutations in the indicated gene.

For the initial diagnostic work-up of AML, the conventional approach of morphologic assessment of the bone marrow remains the universal recommendation by the World Health Organization (World Health Organization [WHO], 2016), the European Leukemia Net (ELN), and the National Comprehensive Cancer Network (NCCN) guidelines (Arber et al., 2016; Döhner et al., 2017; Tallman et al., 2019). The combined information obtained from a battery of tests including flow cytometry, cytogenetics, fluorescence *in situ* hybridization (FISH), and PCR or NGS-based molecular analyses, primarily performed on the bone marrow sample, guides prognostication as well as therapeutic choices for individual patients. The current practice for the evaluation of both remission and MRD for response assessments rely on bone marrow sampling, whereas peripheral blood evaluation may be adequate for monitoring of relapse in most cases (Komanduri and Levine, 2016; Percival et al., 2017).

Once complete clinical remission is achieved post-therapy, the frequency of follow-up bone marrow biopsies is not well defined. Moreover, the clinical utility of monitoring residual leukemic burden during initial induction therapy is currently not known (Wong et al., 2019). Most commonly, once patients attain clinical remission, they undergo repeat bone marrow aspiration every 2 to 3 months during the first year and every 3 to 6 months for the next 2 years as the risk of relapse is maximum during the initial 2 years after the end of consolidation chemotherapy (Percival et al., 2017). A long-standing question remains whether this uncomfortable and inconvenient procedure of bone marrow evaluation can be avoided during certain situations of disease monitoring.

For assurance to a patient in clinical remission to sustain in a clinically disease-free state, several issues remain. These include (i) sampling error introduced from a single bone marrow specimen that represents only a very small fraction of the total bone marrow cellular population (Percival et al., 2017); (ii) spatial heterogeneity and clonal evolution of leukemia (Walter et al., 2012); (iii) occurrence of extramedullary disease at relapse or diagnosis (Ganzel et al., 2016; Solh et al., 2016); (iv) limitation of the sensitivity of routine tests for residual disease monitoring (Ravandi et al., 2018); (v) patient risk stratification; and (vi) monitoring treatment efficacy. Therefore, the search for novel biomarkers that could provide additional predictive and/or prognostic information is ongoing. Subsequent development and validation of these biomarkers may contribute in clinical-decision making and better AML disease management.

## MRD Assessment in AML and Current Challenges

Measurable, molecular, or minimal residual disease (MRD) has emerged as an independent prognostic indicator in AML (Schuurhuis et al., 2018). The current practice for treatment of AML patients post-remission is guided by genetic profile of leukemic blasts at diagnosis and on the MRD-level post-induction and consolidation chemotherapy. The MRD is used as an important tool for early response assessment for prediction of final treatment outcome in AML patients. Unlike the AML NCCN guidelines, the AML European Leukemia Net MRD working group recommends individualized patient-specific reliable tool to monitor MRD for response assessment and risk stratification (Arber et al., 2016; Schuurhuis et al., 2018). Multiparameteric flow cytometry, assessment of fusion transcript levels (i.e., *RUNX1-RUNX1T1*, *CBFB-MYH11*, and *PML-RAR*α) or mutations (*NPM1*, *FLT3* allelic ratio) by quantitative PCR (qPCR) and more recently NGS are commonly used tools for MRD assessment (Jongen-Lavrencic et al., 2018; Voso et al., 2019; Yoest et al., 2020). The ELN is constantly improvising these guidelines for the standardization of methodology and frequency of MRD monitoring.

For MRD assessment, the sample of choice is bone marrow, although, peripheral blood is easy to obtain and lacks immature normal populations of cells that may interfere with analysis by multiparameteric flow cytometry. However, the sensitivity of MRD analysis of the peripheral blood circulating tumor cells (CTCs) is apparently lower than that of bone marrow and requires a lower MRD threshold to be prognostic. Numerous technical challenges remain regarding MRD monitoring in AML including (i) preferred source material (blood versus bone marrow); (ii) ideal sampling time; (iii) sampling interval; (iv) duration of screening for relapse; (v) identifiable and reliable molecular marker/s; and (vi) thorough validation and standardization of MRD assays for each individual marker.

Certain biological concerns include the spectrum of molecular lesions detected in AML clones that predispose them to variable proliferation potential, resulting in temporal difference between the emergence of positive MRD and hematologic relapse. Depending on the AML clone, more frequent MRD monitoring is essential for fast progressors whereas more prolonged MRD monitoring is desirable for slow progressors (Ding et al., 2012). Other confounding factors during MRD analysis include (i) asynchronous development of leukemic clones at different sites; (ii) minor residual leukemic subclones of potentially treatment-resistant nature maybe missed; and (iii) co-occurrence of clonal hematopoiesis of indeterminate potential (CHIP) mutations (e.g., *DNMT3A*, *TET2*, and *ASXL1*) that are associated with age-related clonal hematopoiesis, rather than residual leukemic cells (Busque et al., 2012; Genovese et al., 2014; Jaiswal et al., 2014).

In the rapidly evolving genomic era of precision medicine, a transition towards dynamic monitoring of tumor molecular characteristics is being witnessed. In this regard, easily accessible markers that would reflect the entire heterogeneity of the tumor such as liquid biopsy including CTCs, circulating tumor-derived DNA (ctDNA), plasma cell-free DNA (cfDNA), microRNAs (miRNA), and exosomes have emerged as biomarkers with immense potential (Bronkhorst et al., 2019; Cervena et al., 2019; Silvestri et al., 2020). Plasma cfDNA facilitates non-invasive peripheral blood sampling of tumor-associated actionable alterations that are present in its ctDNA fraction and is gradually finding clinical utility in several cancers. ctDNA has the potential to capture intratumor heterogeneity that maybe missed by tissue biopsy (Bettegowda et al., 2014). An additional advantage of cfDNA analysis may be its utility in cases where the sample quality to perform molecular analysis such as NGS is compromised, with high failure rates. Moreover, integration of cfDNA NGS analysis showed significant increase in the detection of actionable mutations that facilitated molecularly guided therapy (Aggarwal et al., 2019).

## Clinical Studies on the Characterization and Utility of Plasma Cell-Free DNA in AML

Extracellular circulating DNA fragments were initially documented in the human peripheral blood in 1948 (Mandel and Metais, 1948), but its clinical relevance came to light only a few decades later. Circulating DNA was first demonstrated by the presence of anti-dsDNA antibodies detected against a nuclear non-histone substance in the sera of patients with autoimmune disease (Tan et al., 1966). Henceforth, elevated levels of circulating DNA were reported in plasma from cancer patients as compared with healthy controls (Leon et al., 1977) and detectable amounts of cfDNA fragments were also shown in other biofluids (Fleischhacker and Schmidt, 2007). Subsequently, it was demonstrated by Stroun et al. (1989) that only a fraction of the plasma cfDNA was derived from tumor cells characterized by decreased strand stability of cancer cell DNA. This led to the dawn of the concept of liquid biopsy when in 1994, Vasioukhin and Stroun’s collaboration demonstrated that cfDNA carried *N-RAS* point mutations in patients with AML and myelodysplastic syndrome (MDS) (Vasioukhin et al., 1994).

The easy accessibility and usefulness of plasma cfDNA analysis for detection and monitoring of myeloid disorders was subsequently demonstrated by a few promising studies (summarized in Table 2). Preferential detection of *N-RAS* mutations in plasma cfDNA than in DNA obtained from blood cells or bone marrow suggested that a bone marrow biopsy or aspiration may not necessarily contain all the malignant clones involved in the disease (Vasioukhin et al., 1994). These findings were further corroborated by the detection of chromosomal abnormalities, such as loss of heterozygosity and X-chromosome inactivation, preferentially in plasma cfDNA than bone marrow cells obtained from AML patients at diagnosis and post-therapy (Rogers et al., 2004). Furthermore, the alterations of cfDNA levels during the initial phase of induction chemotherapy showed promise as a valuable marker for the early assessment of therapy response and prognostic tool in AML patients (Holdenrieder et al., 2001; Mueller et al., 2006).

**TABLE 2:** Overview of various studies demonstrating the utility of ctDNA for molecular characterization, disease monitoring, and clinical outcome in acute myeloid leukemia.

Study focus	Patients/controls	Molecular method	Gene target	Major findings	Clinical significance	References
Point mutation	10	Southern hybridization/allele-specific PCR	N-RAS	Plasma is easily accessible and useful for detection and monitoring of myeloid disorders	Diagnostic Prognostic	Vasioukhin et al. (1994)
LOH	45/30	Multiplex PCR of microsatellite markers, sequencer	5q, 7q, 8, 17p, 20q	PB plasma is enriched in ctDNA; carry genomic aberrations	Diagnostic	Rogers et al. (2004)
DNA concentration	25	Spectrophotometry	–	Nucleosomal DNA is valuable marker for early prediction of therapeutic efficacy.	Diagnostic Prognostic therapy response	Mueller et al. (2006)
DNA concentration and integrity	60/30	qPCR	*ACTB*	Plasma DNA integrity is increased in acute leukemia and useful for monitoring MRD	Prognostic	Gao et al. (2010)
DNA concentration	66/100	Duplex real-time qPCR		Quantification of plasma DNA is useful for evaluating therapeutic effects and monitoring relapse	Diagnostic Prognostic	Jiang et al. (2012)
Mutation	100	qRT-PCR	*NPM1*	Circulating *NPM* mutations DNA assay serves as a complementary to routine investigative protocol	Diagnostic	Quan et al. (2015)
Gene rearrangement	235	qPCR	IGH or TCR gene rearrangement	Monoclonal IGH and TCR rearrangement in cfDNA may represent a useful tool for MRD monitoring	Prognostic	Zhong et al. (2018)
Mutations	53	NGS, ddPCR	57 targets	ctDNA predicts relapse post-alloSCT in AML and MDS	Diagnostic Prognostic	Nakamura et al. (2019)
Mutations	22	Targeted NGS	28 targets	cfDNA and bone marrow may be complementary in the assessment and monitoring of patients with AML.	Diagnostic Prognostic	Short et al. (2020)

For monitoring MRD and AML disease progression, the changes in the plasma cfDNA concentration and integrity index were shown as potential markers (Gao et al., 2010). Another study demonstrated the clinical utility of plasma DNA quantification by duplex real-time quantitative PCR for the evaluation of therapeutic responses and monitoring relapse in AML patients (Jiang et al., 2012). At diagnosis, the concentration of plasma DNA ranged from 73.4 to 245.1 ng/ml (median value = 168.5), which was significantly higher relative to the control groups and had a male preponderance in patients with AML. Differences in the alterations of plasma cfDNA levels post-chemotherapy between remission and non-remission patients were reported. The documented cfDNA levels in cancer patients ranges between 0 and >1,000 ng/ml of blood (average, 180 ng of cfDNA/ml) and less than 10 to 100 ng/ml (average, 30 ng/ml) in healthy controls (Fleischhacker and Schmidt, 2007). Genetic and epigenetic alterations that are unique characteristics of the tumor of origin can also be analyzed using circulating DNA (Wan et al., 2017; van der Pol and Mouliere, 2019). Indeed, plasma DNA tends to show changes in CpG global methylation early on as compared with peripheral blood cells as shown in patients with MDS (Iriyama et al., 2012).

A limitation of majority of the studies focusing on plasma cfDNA levels was a lack of assessment of tumor mutation burden (TMB), defined as the total number of non-synonymous mutations in the coding regions of the genes. A significant correlation between TMB and efficacy of targeted therapy is emerging. The clinical utility of assessing TMB using plasma cfDNA, which contains ctDNA, as a surrogate specimen to biopsy has been proposed (Cao et al., 2019; Fancello et al., 2019). To investigate the association of circulating *NPM1* (a tetra nucleotide duplication of TCTG in exon 12) mutation levels with clinical characteristics of AML patients, researchers quantitated the copies per milliliter of *NPM1*-A mutation in plasma cfDNA (Quan et al., 2015). This group demonstrated that the plasma is indeed enriched with tumor DNA and suggested the utility of monitoring *NPM1*-mutated AML using circulating DNA as a complementary assay to the routine molecular protocols. Furthermore, the utility of plasma ctDNA was demonstrated by detection of monoclonal IGH and TCR rearrangement for MRD monitoring in patients with AML (Zhong et al., 2018). During follow-up, recurrence of these rearrangements in cfDNA was observed 1-3 months earlier than bone marrow relapse indicating cfDNA as a useful tool for MRD monitoring in patients with AML.

The technological advancement in the genomic profiling of ctDNA facilitated the tracking of driver mutations and karyotypic abnormalities in AML and MDS for disease monitoring and assessment of treatment response. Highlights of the co-evolution of important breakthroughs in AML genomics and utility of cfDNA analysis are shown in Figure 1. A customized 55 genes panel for targeted deep sequencing of known recurrent mutations in MDS and AML was used for ctDNA analysis to monitor therapeutic response and clonal evolution in MDS (Yeh et al., 2017). These findings supported that ctDNA dynamics correlated with tumor burden during therapy for MDS. In another retrospective study, the utility of ctDNA analysis was evaluated for the identification of high-risk AML/MDS patients for relapse post-myeloablative allogeneic stem cell transplantation (alloSCT) (Nakamura et al., 2019). Nakamura et al. (2019) identified the driver mutations by NGS in each sample at diagnosis, which were then tracked using ctDNA analysis using personalized droplet digital PCR (ddPCR) for MRD quantification. Promisingly, ctDNA analysis-based MRD positivity in a few MRD-negative patients by conventional bone marrow sampling suggested the potential of ctDNA as a better representative of residual AML in these patients. The benefit of ctDNA-based MRD positivity especially in cytopenic patients after alloSCT could predict relapse within a month after alloSCT and correlated with shorter overall survival. Although, this approach is potentially limited by the requirement for a unique digital PCR assays for each alteration and therefore is most suitable for recurrent mutations (*IDH1/2* genes and hotspot mutations in other genes).

Sequencing of cfDNA may identify clinically relevant mutations not detected in the bone marrow and may play a role in the assessment of MRD and prediction of relapse as further corroborated in a recent study. Targeted NGS of plasma cfDNA and bone marrow at the time of diagnosis and after achieving remission was conducted in 22 patients with AML (Short et al., 2020). Among 28 genes sequenced, a total of 39 unique somatic mutations were detected. Five mutations (13%) were detected exclusively in cfDNA, and 15 (38%) were detected only in the bone marrow. Among the 19 mutations detected in both sources, high concordance of variant allelic frequency (VAF) was observed by both methods although, sequencing of cfDNA detected new or persistent leukemia-associated mutations during remission that predicted relapse. It appears that when the VAF was <10%, either method may miss small subclonal populations suggesting that cfDNA and bone marrow analyses may be complementary in the assessment and monitoring of patients with AML. It is recommended that ctDNA may therefore be examined by error-corrected NGS, and the most convenient and reproducible approach will need to be established by individual laboratories as described in the next section (Rolfo et al., 2020).

The studies so far demonstrate a great potential for ctDNA in AML MRD assessment and thus may relieve patients from frequent risk-prone painful bone marrow punctures. Monitoring ctDNA enhances detection of relapse and defines molecular remission better. Early intervention at the stage of minimal tumor burden maybe possible by serial monitoring of ctDNA. Indeed, our experience with longitudinal molecular monitoring of plasma ctDNA during the course of AML disease progression supports these findings as described below.

## Case Study

**FIGURE 2:**
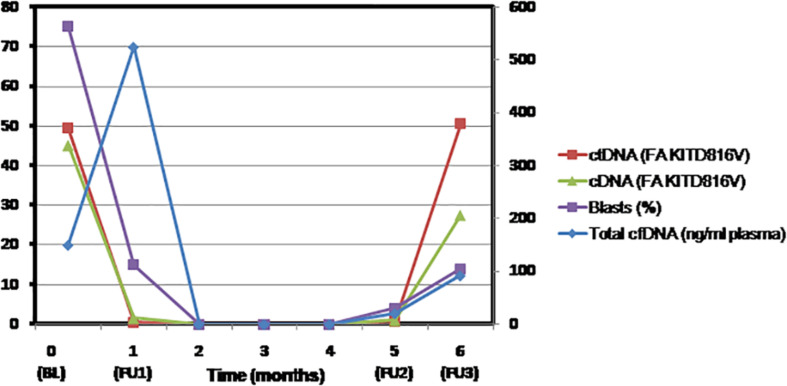
The fractional abundance of *KIT* mutation in circulating tumor-derived DNA but not the levels of plasma cell free DNA represent residual AML better and may predict early relapse. Molecular monitoring of plasma cell-free DNA and bone marrow samples of AML patient was performed by droplet digital PCR at the indicated time points. Shown in the line graph are blast percentage (violet square), total plasma cfDNA concentration in nanograms per milliliter plasma (blue diamond), fractional abundance of *KIT* D816V mutation in cfDNA (red square), and bone marrow (green triangle) at baseline (BL = Day 0), MRD assessment (FU1 = Day 30), clinical remission (FU2 = 5 months), and after consolidation and relapse (FU3 = 6 months). BL, baseline; cDNA, complementary DNA; cfDNA, cell-free DNA; ctDNA, circulating tumor-derived DNA; FA, fractional abundance; FU, follow-up; MRD, measurable residual disease.

In a case of AML with *RUNX1-RUNX1T1* recurrent abnormality and *KIT* D816V mutation, we retrospectively determined the fractional abundance of *KIT* D816V mutation by droplet digital PCR. Paired samples of plasma cfDNA and bone marrow aspirates were collected at baseline, for MRD assessment, at CR after consolidation and relapse (Supplementary Data). A strong concordance was observed between the fractional abundance of *KIT* mutation but not the plasma cfDNA levels in both plasma and bone marrow samples at various indicated time points (Figure 2). Indeed, at relapse, the VAF of *KIT* mutation was represented better in the plasma cfDNA indicating it as a superior method for improved detection of persistent leukemia-associated mutation during remission that may predict early relapse. The higher turnover rate of leukemic cells than normal cells possibly contributes to the elevated cfDNA levels into circulation as was evident by increase in the levels of cfDNA post-induction. Therefore, mutation burden seems to be a more reliable marker for early treatment response than the plasma cfDNA levels. It is suggested that more frequent monitoring of ctDNA for the persistent mutations would allow an early prediction of relapse. Nevertheless, our findings need to be validated in a larger cohort.

## Current Status of Ultrasensitive Technology Platforms for Genotyping ctDNA

The ctDNA accounts for <1% of the total cfDNA, which is a concoction of DNA fragments released by necrosis, apoptosis, and active secretion (Bettegowda et al., 2014; Diaz and Bardelli, 2014; Kustanovich et al., 2019). Depending on the disease burden, cellular turnover, stage, and treatment response, the detection and quantification of small quantities of ctDNA is challenging specially with low disease burden where the total amount of ctDNA might be 0.01% of the total cfDNA (Diehl et al., 2008; Forshew et al., 2012; Bettegowda et al., 2014; Kennedy et al., 2014; Newman et al., 2014; Gorgannezhad et al., 2018). With the currently available technologies for ctDNA characterization, a targeted or an untargeted approach may be used depending on the tumor burden. The targeted strategy is useful for tracking specific tumor mutations detected at the time of disease diagnosis at baseline (Siravegna et al., 2019; Cristofanilli and Braun, 2010). The untargeted methods focus on whole genome or exome sequencing that may detect copy number alterations or novel point mutations (Elazezy and Joosse, 2018; Gorgannezhad et al., 2018).

Various ultrasensitive technologies for detection of ctDNA include qPCR, digital PCR, targeted NGS, and mass spectrometry. These techniques offer both high sensitivity (VAF as low as ∼0.01%) and specificity (Cristofanilli and Braun, 2010; Freidin et al., 2015). Technological advances in real-time qPCR have made it possible to detect mutations with <10% VAF with higher sensitivity. This approach utilizes enrichment of common variant alleles by preferential amplification by blocking amplification at the oligo 3′ end (Elazezy and Joosse, 2018). Droplet digital PCR and Beads, Emulsions, Amplification, and Magnetic (BEAMing) technologies are two common digital platforms for liquid biopsy, which may detect mutant alleles with a high sensitivity of 0.001–0.01% (Vogelstein and Kinzler, 1999; Kristensen and Hansen, 2009). The principle of droplet digital PCR is based on water-oil emulsion droplet technology. Both the mutated and wild-type DNA samples are partitioned into thousands of droplets, and PCR amplification of each template occurs in individual droplets. Real-time PCR fluorescent-specific probes can be used for the detection of fluorescent-positive and fluorescent-negative droplets for quantitation of the absolute DNA copies using ddPCR (Kristensen and Hansen, 2009). This methodology is limited by the number of genomic targets that can be multiplexed. Another important technique is BEAMing that combines emulsion PCR, magnetic separation of the PCR product, hybridization with base-pair–specific fluorescent probes to distinguish wild-type and mutant alleles, and flow cytometry to identify and quantify specific somatic mutations present in the DNA (Thress et al., 2015). Although, digital PCR offers cost-effective platform as compared with NGS, a unique assay needs to be designed for each mutation whereas NGS allows evaluation of multiple genomic aberrations simultaneously.

**FIGURE 3:**
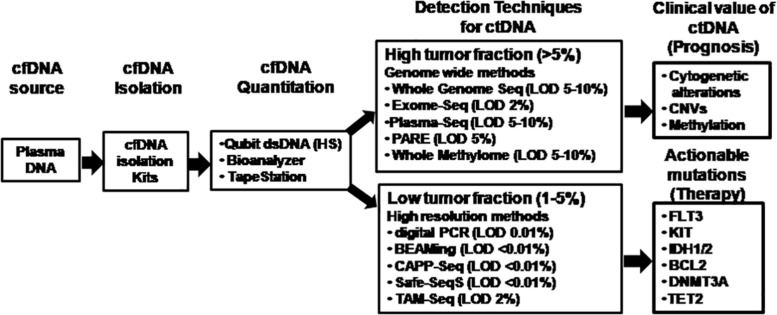
Comparison of technology platforms for analysis of cell-free DNA. The analytical workflow for cell-free DNA characterization and analysis are depicted showing key steps of isolation, quantitation, and various options of the technology platforms available for molecular analysis of circulating tumor-derived DNA depending on the target region and tumor burden. cfDNA, cell-free DNA; ctDNA, circulating tumor-derived DNA; CAPP-Seq, cancer personalized profiling by deep sequencing; CNVs, copy number variations; dsDNA (HS), double-stranded DNA (high sensitivity); LOD, limit of detection; PARE, parallel analyses of RNA ends; Safe-SeqS, safe-sequencing system; Tam-Seq, tagged amplicon deep sequencing.

The major technical revolution witnessed is the range of ultrasensitive NGS platforms available for optimized detection of multiple somatic alterations simultaneously using ctDNA. The choice of target regions to be identified governs the selection of the NGS platform (Figure 3). Ultra-Deep NGS allowed quantitation of low-abundance DNA variants with high coverage in ctDNA by using error suppression multiplexed deep sequencing strategy with a limit of detection of approximately 0.02% (Narayan et al., 2012; Uchida et al., 2015). Several protocols including safe-sequencing system (Safe-SeqS) (Kinde et al., 2011), tagged amplicon deep sequencing (Tam-Seq) (Forshew et al., 2012), cancer-personalized profiling by deep sequencing (CAPP-Seq) (Newman et al., 2014), bias-corrected targeted NGS (Paweletz et al., 2016), and multiplex PCR NGS (Abbosh et al., 2017) have been evaluated. Safe-SeqS is based on assignment of unique identifiers to prespecified DNA templates, amplification of each tagged region into families and sequencing of the amplification products (Kinde et al., 2011). Tam-Seq is a protocol optimized for low-frequency hotspot mutations in circulating DNA at allele frequencies as low as 2%, with sensitivity and specificity of >97% (Forshew et al., 2012). CAPP-Seq is a capture-based NGS method that enriches for defined genomic regions prior to sequencing by hybridization of target regions to antisense oligonucleotides with a theoretical detection limit of 0.00025% VAF (Newman et al., 2014). Bias-corrected targeted NGS uses multifunctional markers including sample and sequence tags that bind to small capture probes which are amplified with high specificity (Paweletz et al., 2016). Finally, multiplex-PCR NGS is the combination of targeting clonal and subclonal single nucleotide variants selected to track phylogenetic tumor branches in plasma by high-throughput PCR amplification and sequencing reaching 99% sensitivity for the detection of mutations in the target regions (Abbosh et al., 2017).

Amplification-based methods including PCR and NGS are limited by the need for specific primers to initiate the amplification process. In this scenario, the utility and flexibility of HPLC-based mass spectrometry methods for the analysis of short DNA sequences is promising (Sharma et al., 2011). The short oligonucleotide mass analysis combines PCR amplification, restriction digestion, and electrospray ionization mass spectrometry and is more sensitive than restriction fragment length polymorphism-PCR for detection of specific mutations from circulating cfDNA. The more popular technique is matrix-assisted laser desorption ionization time-of-flight mass spectrometry (MALDI-TOF MS) for analyzing point mutations in cfDNA samples. Moreover, quantitative analysis of DNA methylation based on unique masses of methylated and unmethylated products by MALDI-TOF-MS MassARRAY system detects CpG sites.

## Applications OF ctDNA Analysis in AML

**FIGURE 4:**
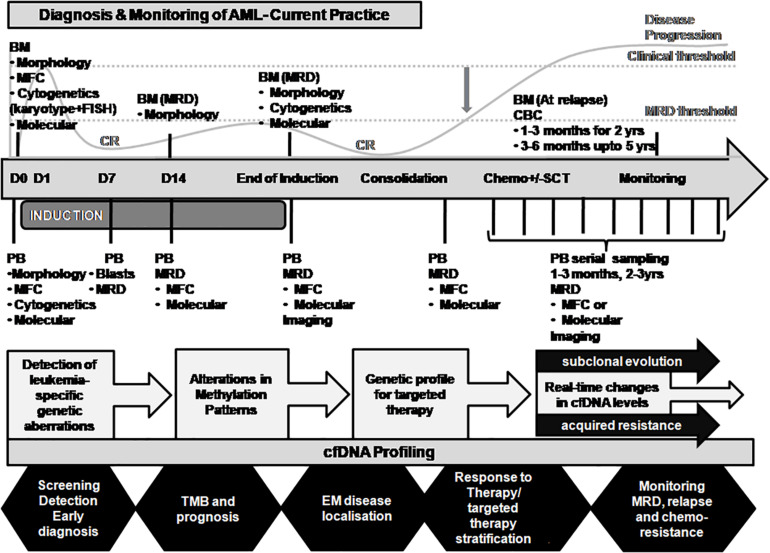
Proposed integration of ctDNA profiling with AML diagnosis and disease monitoring. The current practice for AML diagnosis and disease monitoring is primarily dependent on the bone marrow sampling. A representation of the disease course is merged with the major time-points of investigation for evaluation of response to treatment (top panel). A critical time point post-chemotherapy needs to be defined when the disease is in occult stage and the MRD threshold is maintained (indicated by the gray downward arrow). Residual AML disease monitoring by serial sampling of circulating cfDNA analysis for patient-specific genetic alterations may prove informative when MRD threshold is breached (indicated in the middle panel). cfDNA profiling may provide several other applications in AML disease monitoring in real time as shown (bottom panel). BM, bone marrow; CBC, complete blood count; cfDNA, cell-free DNA; CR, clinical remission; MFC, multiparametric flow cytometry; MRD, measurable residual disease; PB, peripheral blood; SCT, stem cell transplantation; TMB, tumor mutation burden.

The applications of ctDNA analysis go beyond initial diagnosis, treatment stratification and monitoring, prognosis, assessment of MRD, and prediction of relapse (Mader and Pantel, 2017; Wan et al., 2017; Corcoran and Chabner, 2018; Heitzer et al., 2019; Pantel and Alix-Panabières, 2019; Cescon et al., 2020). cfDNA is an ideal non-invasive tool that allows periodic multiple tests over time and provides real-time data on tumor dynamics and evolution. Furthermore, longitudinal monitoring of ctDNA allows tracking of both genomic and epigenomic alterations (van der Pol and Mouliere, 2019; Erger et al., 2020). In addition to its use in personalized medicine, ctDNA analysis holds considerable promise as a surrogate marker for several applications such as (i) cancer screening in asymptomatic individuals (Chen and Zhao, 2019); (ii) early detection of cancer occurrence (Chen and Zhao, 2019); (iii) tumor localization and staging (van der Pol and Mouliere, 2019); (iv) monitoring of clonal evolution of malignant cells in real-time (De Mattos-Arruda et al., 2014; Fittall and Van Loo, 2019); (v) longitudinal ctDNA dynamics reflect tumor burden during treatment through serial sampling; (vi) identification of acquired drug resistance mechanisms (Russo and Bardelli, 2017; Diao et al., 2020); (vii) representative sampling of unreachable and non-resectable cancers; and (viii) utility in clinical trials (Ulrich and Paweletz, 2018; Araujo et al., 2019; Cescon et al., 2020). Potential integration of real-time ctDNA profiling and its applications in AML disease monitoring are depicted in Figure 4.

## Current Challenges in cfDNA Analyses

The International Society of Liquid Biopsy (ISLB) has recently elaborated on the various challenges associated with cfDNA analysis such as technological, biological, reporting-related, socio-economic, and regulatory (Rolfo et al., 2020). It is established that the plasma is a preferred choice because of the genomic DNA contamination in the serum (Steinman, 1979). The technological challenges primarily deal with preanalytical and analytical considerations which have been described earlier by our group (Thakral et al., 2020) and others (Merker et al., 2018; Volckmar et al., 2018; Kerachian et al., 2019; Li et al., 2019; Geeurickx and Hendrix, 2020; Rolfo et al., 2020). The sensitivity of cfDNA analysis is dependent on several variables from sample collection and processing, for which recommendations have been proposed to circumvent these issues (Rolfo et al., 2020). Since cfDNA levels alter during the course of the disease and are influenced by treatment, therefore, the dynamics of cfDNA can be potentially used as a real-time monitoring method (Malapelle and Rolfo, 2020). However, because of low abundance of cfDNA in certain cases, alternate methodologies are being developed for improved detection of genetic alterations such as analysis of circulating tumor RNA (ctRNA) for evaluation of fusion transcripts (Benayed et al., 2019).

One of the primary biological challenges in context of AML is CHIP. Presence of specific recurrent genetic variants (e.g., *DNMT3A*, *TET2*, and *ASXL1*) drive asymptomatic proliferation of blood cells arising from hematopoietic stem cells harboring them. These age-related CHIP mutations become a potential source of false positives in cfDNA in the absence of a hematological malignancy (Busque et al., 2012; Genovese et al., 2014). As mentioned earlier, their presence poses a challenge for MRD assessment as well as early cancer detection. To overcome this issue, a novel CHIP-filtering approach has been evaluated recently that is based on whole blood cell sequencing and cfDNA analysis with a sensitivity equivalent to droplet digital PCR (Abbosh et al., 2019; Liu et al., 2019). Another issue that might be applicable to extramedullary disease in AML is that of cfDNA shedding (Solh et al., 2016). As the sites of disease progression influence the cfDNA levels and may result in false negativity, this needs to be investigated further.

Till date, no universal consensus exists on the minimal requirement of NGS panels or their cost-effectiveness and therefore most commercial or customized platforms attempt to incorporate detection of genetic alterations covering actionable targets. A limitation of such platforms designed using hotspots alone is that they tend to miss on novel mutations. It is recommended to use a broader gene panel that allows improved stratification of patients for clinical trials and repurposing of FDA-approved drugs. With regard to AML, detection of gene fusions maybe challenging to detect using cfDNA because of the variability in the available hybrid capture techniques. Moreover, vendor and reporting related issues remain that commercially available plasma NGS platforms should be used with caution in clinical practice as indiscriminate use of clinical suggestions of FDA-approved drugs, clinical trials, and bioinformatics based-therapies is indicated. It is recommended to evaluate the information so obtained in context of a molecular tumor board and use of standardized criteria to define actionability through the use of evidence-based scales such as OncoKB (Chakravarty et al., 2017) and ESCAT (Mateo et al., 2018).

A rigorous validation is mandatory before clinical utilization of cfDNA-based assays. To accelerate this process, a two-tier system has been created, one for the development and validation of assays (Blood Profiling Atlas in Cancer aka Blood PAC) and a regulatory body the International Liquid Biopsy Alliance (ILBA) that includes ISLB and regulatory agencies with a goal of increasing efficiency, minimizing duplication and expedition of the inclusion of liquid biopsy based assays into routine clinical practice. So far, the only FDA-approved liquid biopsy tests include Cobas EGFR mutation assay for non-small-cell lung carcinoma (Kwapisz, 2017) and Therascreen PIK3CA RGQ mutation detection assay in breast cancer.

## Future Direction and Conclusion

Liquid biopsy technologies for qualitative and quantitative analysis of cfDNA have immensely broadened our ability to assess cancer, specifically in its cryptic stage without the need for invasive sampling. A serial longitudinal monitoring of AML using cfDNA analysis from the peripheral blood may provide a series of screenshots that can potentially be coalesced together to generate a time-lapse movie of clonal evolution of leukemia. With an almost excellent concordance with matched bone marrow aspirates, cfDNA can quantify disease burden by providing mutational landscape as well as identify acquired drug resistance mechanisms which may guide clinical management in real time.

As rapid diagnosis of genetic alterations is required to opt for appropriate induction therapy by the oncologists, therefore, a quick turnaround time for comprehensive cfDNA sequencing may provide actionable information to guide clinical management. In this regard, the convenience of sample acquisition and storage of cfDNA provides added procedural advantage over currently practiced protocols. So far, the small cohorts evaluated for cfDNA necessitates further validation with a certain sample size to examine the frequency of the gene mutations in AML patients that may facilitate significant statistical power for meaningful comparisons. Initiatives have been taken by researchers through multicentric studies, cooperative-group trials, and regulatory bodies are formulated to devise universal guidelines. Further investigations will have to define the role of cfDNA as a diagnostic tool by determining clinically relevant tumor thresholds relative to currently used routine parameters.

The use of ultrasensitive sequencing technologies has facilitated the reliable detection of very low amounts of ctDNA that excellently complement the currently used methods. Therefore, an immediate clinical application of cfDNA genotyping in AML may be its incorporation in larger prospective clinical trials for the identification of patients carrying actionable mutations and their longitudinal genetic monitoring during targeted therapy administration. Monitoring of ctDNA may provide surrogate end-points for clinical outcomes. This can reduce the extended follow-up durations as well as become more cost-effective for clinical trials related to adjunct therapies. Moreover, ctDNA analyses based MRD estimation may aid the discovery of novel drugs targeted at elimination or control of residual tumor in high-risk AML patients prone to relapse (Pantel and Alix-Panabières, 2019).

Plasma ctDNA may be used for the study of virtually all genomic abnormalities including both coding and non-coding regions, loss of heterozygosity, microsatellite loci, mutations, polymorphisms, methylation, and copy number variations. Deep sequencing as a standalone for ctDNA analyses may get a boost by additional assessment of epigenetic modifications, immune signatures, platelets, exosomes, and miRNA analyses. Moreover, a better understanding of the dynamics of ctDNA in AML disease progression may be necessary before blood can be routinely used as a source to monitor tumor burden. Nevertheless, longitudinal monitoring of AML patients by ctDNA analysis could complement bone marrow cells as a diagnostic marker without sampling bias.

## Author Contributions

DT and RG designed and conducted the study, performed the data interpretation, statistical analysis, and wrote the manuscript. RKS is the medical oncologist who treated the patient included in the case study. PV performed the ddPCR experiments. IK performed NGS experiments. SV conducted RT-PCR and fragment analysis assays. All authors contributed to the article and approved the submitted version.

## Conflict of Interest

The authors declare that the research was conducted in the absence of any commercial or financial relationships that could be construed as a potential conflict of interest.

## Abbreviations

AML: acute myeloid leukemia
AlloSCT: allogeneic stem cell transplantation
CAPP-Seq: cancer personalized profiling by deep sequencing
CHIP: clonal hematopoiesis of indeterminate potential
cfDNA: cell-free DNA
ctDNA: circulating tumor-derived DNA
CpG: cytosine and guanine dinucleotides
CN-AML: cytogenetically normal karyotype AML
CTCs: circulating tumor cells
ddPCR: droplet digital PCR
dsDNA: double-stranded DNA
EGFR: epidermal growth factor receptor
ELN: European Leukemia Net
FDA: Food and Drug Administration
FISH: fluorescence *in situ* hybridization
HPLC-MS: high performance liquid chromatography based mass spectrometry
IGH: immunoglobulin heavy chain
ISLB: International Society of Liquid Biopsy
MALDI-TOF MS: matrix-assisted laser desorption ionization time-of-flight mass spectrometry
MDS: myelodysplastic syndrome
MRD: measurable residual disease (also known as molecular or minimal residual disease)
NCCN: National Comprehensive Cancer Network
NGS: next-generation sequencing
PCR: polymerase chain reaction
qPCR: quantitative PCR
Safe-SeqS: safe-sequencing system
Tam-Seq: tagged amplicon deep sequencing
TCR: T cell receptor
TMB: tumor mutation burden
VAF: variant allelic frequency
WHO: World Health Organization.
