# Supplementary material for: Real-Time Molecular Monitoring in Acute Myeloid Leukemia With Circulating Tumor DNA
Source: Front Cell Dev Biol. 2020 Dec 11;8:604391. doi: 10.3389/fcell.2020.604391 (PMC7759522; doi:10.3389/fcell.2020.604391)
Supplement: Supplementary file 1 [file Table_1.DOCX]

**(Supplementary data)**

**Case study**

A 28 year old male patient presented with high grade fever, multiple joint pain, breathlessness, and episodic vomiting and severe anorexia, on evaluation, was found to have anemia, thrombocytopenia, and a white blood cell count (WBC) of 15900/mm^3^. Cellular bone marrow aspirate showed near total replacement by blasts and morphology. Flow cytometric immunophenotyping showed 75% CD45dim blasts with high side scatter which were CD34dim+, CD117+, CD13+, CD33+ cMPOdim, CD38+, HLA-DR+(htg), CD56+, CD4dim, CD64dim and negative for cCD79a, CD19, CD3, CD7, CD16, CD11b, CD123, CD36, and CD14 indicating AML-M2. Cytogenetics revealed trisomy of chromosome 4 along with t(8;21)(q22;q22) which was corroborated by a positive RT-PCR for *RUNX1-RUNX1T1*. Targeted sequencing by the Trusight myeloid panel of the genomic DNA sample obtained from leukemic cells at baseline revealed *cKIT D816V* mutation, hence assigning an intermediate risk category. The patient was admitted for 3+7 induction (Daunorubicin 100mg D1 and D2; 65mg on D3 and ARA-C 200mg D1-D3 and 100mg D4-D7). On D25 post induction, the patient was administered 1 unit PRBC transfusion along with antibiotics. Since the patient did not achieve CR after 3+7 induction so he was given reinduction with HiDAC (18g/m^2^) followed by 2 rounds of HiDAC consolidation which was well tolerated and also planned for Dasatanib (100mg OD) and achieved CR. The patient relapsed after six months and was given ADE induction (Ara-C 150mg D1-D10; Daunorubicin 75mg D1, D3, D5 and Etoposide 150mg D1-D5). After a month of ADE induction patient succumbed to febrile neutropenia and was managed with IV antibiotics. The patient had refractory AML and was maintained on Venetoclax.
